# Clinical Validity of FoundationOne Liquid CDx for Detection of *BRAF*^V600E^ in Colorectal Cancer

**DOI:** 10.1158/2767-9764.CRC-25-0002

**Published:** 2025-09-09

**Authors:** Rona Yaeger, Jean-François Martini, Lincoln Pasquina, Brian Tunquist, Xiaosong Zhang, Fatima Kaiser, Norberto Pantoja Galicia, Shibing Deng, Siliang Gong, Cui Guo, Jimmy Kiely, Ta-Chou Vincent Ng, Graham Ferrier, Josep Tabernero, Scott Kopetz

**Affiliations:** 1 Department of Medicine, Memorial Sloan Kettering Cancer Center, New York, New York.; 2 Translational Science Operations, Pfizer, Inc., La Jolla, California.; 3 Clinical Development, Foundation Medicine Inc., Boston, Massachusetts.; 4 Formerly Pfizer, Inc., New York, New York.; 5 Global Development, Pfizer, Inc., South San Francisco, California.; 6 Formerly Foundation Medicine Inc., Boston, Massachusetts.; 7 Biometrics, Foundation Medicine Inc., Boston, Massachusetts.; 8 Global Medical, Pfizer, Inc., New York, New York.; 9 Department of Medical Oncology, Vall d’Hebron Hospital Campus and Vall d’Hebron Institute of Oncology (VHIO), University of Vic – Central University of Catalonia, Barcelona, Spain.; 10 Department of Gastrointestinal Medical Oncology, University of Texas MD Anderson Cancer Center, Houston, Texas.

## Abstract

**Purpose:**

The BRAF inhibitor encorafenib (Enco) plus the anti-EGFR antibody cetuximab (Cetux) improved overall survival, objective response rate, and progression-free survival in previously treated *BRAF*^V600E^-mutant metastatic colorectal cancer in BEACON, a phase III randomized trial, leading to regulatory approval for this indication. To support rapid, plasma-based testing for *BRAF*^V600E^ identification, clinical validity of a ctDNA-based assay, FoundationOneLiquid CDx (F1LCDx), was assessed against the reference tumor-based clinical trial assay (CTA) in liquid biopsy–evaluable samples from BEACON and commercially obtained tissue-matched plasma samples.

**Patients and Methods:**

Pretreatment tissue samples were collected in BEACON to confirm *BRAF* mutational status using the central single gene PCR assay. Concordance between the CTA and liquid biopsy tests was assessed, and clinical validity of liquid biopsy testing was examined using clinical outcomes from BEACON.

**Results:**

Of the 523 evaluable patients, 433 with matched tissue and plasma samples had CTA and F1LCDx results available (BEACON, *n* = 328; commercial, *n* = 105). A strong concordance in detecting *BRAF*^V600E^ was found between F1LCDx and CTA, with a positive percent agreement of 87.2% and negative percent agreement of 97.1%. Among 42 F1LCDx−/CTA+ samples, 41 (97.6%) had ctDNA tumor fraction <1%. Among samples with ctDNA tumor fraction >1%, the positive percent agreement was 99.4% and negative percent agreement was 86.7%. Clinical outcomes with Enco plus Cetux were similar between those identified as F1LCDx+/CTA+ and CTA+ overall.

**Conclusions:**

This study supports using liquid biopsies as a clinically valid assay for identifying *BRAF*^V600E^ alterations in patients with metastatic colorectal cancer, particularly when ctDNA tumor fraction was >1%.

**Significance:**

In the phase III BEACON trial, which established Enco plus Cetux as a standard of care for previously treated *BRAF*^V600E^-mutant metastatic colorectal cancer, mutational status was confirmed through testing of tumor tissue. To support rapid, less invasive testing for *BRAF*^V600E^ in plasma, this retrospective study assessed a ctDNA-based assay and found strong concordance between the liquid biopsy test and the tumor-based assay in detecting *BRAF*^V600E^.

## Introduction

Advances in detection of ctDNA have made liquid biopsy (LBx) an increasingly utilized method for tumor profiling (1). ctDNA-based tumor profiling is less invasive and has a shorter turnaround time than tissue-based DNA profiling, which is critical for patient diagnosis and management. In addition, LBx allows for multiple serial sample collections, which can be useful for reflecting real-time tumor information, detecting disease progression, monitoring treatment response, and identifying potential biomarkers of resistance or sensitivity (2, 3). In gastrointestinal cancer trials, ctDNA genotyping has resulted in significantly reduced screening time and improved trial enrollment without compromising treatment efficacy compared with tissue genotyping (4). In recent years, several ctDNA-based companion diagnostic assays, including FoundationOne Liquid CDx (F1LCDx), cobas EGFR Mutation Test v2, and Guardant360 CDx, were approved by the US FDA to detect biomarkers in patients with non–small cell lung cancer (5). Although the treatment of metastatic colorectal cancer (mCRC) often involves resection which allows tissue availability, detection of targetable alterations by LBx can be a clinically desirable and less invasive disease-monitoring option after first-line treatment (6). Although archived tissue biopsy only reflects static tumor information, LBx can reflect real-time tumor information and allows dynamic monitoring of mutations during disease progression (3). mCRC is among the most common tumor types for detectability of ctDNA (7, 8), making LBx a particularly attractive option for tumor profiling. However, ctDNA-based assays vary in genes tested, methodology, and sensitivity (9).

The BEACON trial was a randomized, open-label, multicenter, three-arm, phase III study that compared the efficacy and safety of encorafenib (Enco; Braftovi) + binimetinib (Mektovi) + cetuximab (Cetux; Erbitux) and the Enco + Cetux arm with irinotecan/Cetux or FOLFIRI/Cetux (control arm) in patients with a *BRAF*^V600E^ alteration in colorectal cancer whose disease had progressed after one or two prior regimens in the metastatic setting (10). The clinical trial tested the efficacy of therapy, with enrollment based on tissue-based PCR and next-generation sequencing (NGS)–based local assay results with confirmation by clinical trial assay [CTA; the investigational Qiagen Therascreen BRAF V600E RGQ PCR Kit (Qiagen)] or enrollment through CTA to select patients with colorectal cancer whose tumors harbor the *BRAF*^V600E^ alteration. Enco in combination with Cetux is the first and only FDA-approved targeted therapy regimen for the treatment of adult patients with mCRC with a *BRAF*^V600E^ mutation after prior therapy based on the efficacy population of the Enco + Cetux arm (*n* = 220) versus the control arm (*n* = 221). Overall survival (OS) and objective response rate (ORR) by RECIST version 1.1 were the major efficacy outcomes, and the median progression-free survival (PFS) and duration of response were additional efficacy outcome measures. For patients treated in the Enco + Cetux arm of the study, the median OS was 8.4 months [95% confidence interval (CI), 7.5–11.0] and the median PFS was 4.2 months (95% CI, 3.7–5.4). ORR (20%; 95% CI, 13%–29%) and duration of response (6.1 months; 95% CI, 4.1–8.3) were both assessed in the subset of the first 220 patients who were included in the randomized portion of the Enco + Cetux (*n* = 113) and control (*n* = 107) arms.

The Qiagen Therascreen assay is a real-time PCR *in vitro* diagnostic device for the qualitative detection of the *BRAF*^V600E^ alteration in patients with mCRC. The assay detects the V600E alteration in the *BRAF* gene using genomic DNA extracted from formalin-fixed, paraffin-embedded colorectal cancer tissue. The FDA granted pre-market approval to the Qiagen Therascreen for use as a companion diagnostic to identify patients with mCRC whose tumors harbor the *BRAF*^V600E^ alteration to be treated with Enco in combination with Cetux (11). F1LCDx is a qualitative NGS-based *in vitro* diagnostic test that uses targeted high-throughput hybridization–based capture technology to detect and report substitutions, insertions, and deletions in 311 genes. F1LCDx utilizes circulating cell-free DNA (cfDNA) isolated from plasma derived from anti-coagulated peripheral whole blood collected from patients with cancer and is intended to be used as a companion diagnostic to identify patients who may benefit from treatment with the targeted therapies (12). The F1LCDx test is the first LBx companion diagnostic assay approved by the FDA that offers comprehensive tumor profiling to identify *BRAF*^V600E^ alterations in patients with mCRC who are eligible for treatment with Enco in combination with Cetux (13).

This clinical bridging study evaluated the concordance between CTA and F1LCDx and the clinical efficacy of Enco in combination with Cetux in patients with mCRC harboring the *BRAF*^V600E^ mutation identified by F1LCDx, through retrospective analysis of banked plasma samples from patients enrolled in the BEACON study. In addition, in a *post hoc* exploratory analysis not prespecified in the clinical bridging statistical analysis plan, we further investigated the sensitivity of F1LCDx as a function of ctDNA content as measured by ctDNA tumor fraction (TF).

## Materials and Methods

### BEACON ethical statement

The BEACON trial was approved by the institutional review board or independent ethics committee at each center and was conducted in accordance with the requirements of the regulatory authorities of each country and with the provisions of the Declaration of Helsinki and the Good Clinical Practice guidelines of the International Council on Harmonization (10). All patients provided written informed consent.

### Clinical bridging study design and samples

The BEACON CTA was performed on tumor tissue collected any time prior to trial enrollment using the Qiagen Therascreen *BRAF* V600E RGQ PCR Kit. This study was conducted to evaluate the concordance between the CTA and a CTA based on F1LCDx (LBx test) in identifying patients with mCRC harboring *BRAF*^V600E^ from the Enco + Cetux and control arms. Based on this, the efficacy in the Enco + Cetux arm versus the control arm in patients identified as *BRAF*^V600E^-positive by the LBx test was estimated, and the robustness of concordance analysis and efficacy analysis was assessed with a sensitivity analysis that accounts for the uncertainty due to missing data for the *BRAF*^V600E^ alteration status as defined by the LBx test.

Pretreatment plasma samples were collected at the time of trial screening from participants in the BEACON trial by the therapeutic investigational sites per the study protocol and shipped to the College of American Pathologists–/Clinical Laboratory Improvement Amendments–certified central testing laboratories. LBx testing was performed on available plasma samples from patients in the Enco + Cetux and control arms in the BEACON clinical trial who tested positive for *BRAF*^V600E^ by CTA (CTA+). Additionally, commercially procured *BRAF*^V600E^-negative tissue samples from patients with colorectal cancer with matched plasma were tested. The LBx test clinical bridging study involved retrospective testing of plasma samples from the BEACON clinical trial; as such, no additional patient follow-up was conducted.

Assessment of detection on F1LCDx was performed without knowledge of the CTA results. The analysis plan was created without visibility into clinical information. Assessment of concordance was performed as prespecified, independent of the clinical information. Clinical information was available during the assessment of clinical validity. For the Qiagen Therascreen test, the limit of detection (LoD) for the *BRAF*^V600E^ system is 7.8% [mutant allele frequency (MAF)], which represents the highest LoD value observed across all DNA inputs; however, with high DNA input, the LoD is 2% MAF (14). For F1LCDx, the LoD (the lowest MAF at which 95% of target variants are detected) for *BRAF*^V600E^ in colorectal cancer samples is 0.33% MAF (15).

Inclusion criteria for samples included in this clinical bridging study were specimens that were in frozen plasma, met the minimum criteria for LBx operational testing requirements, and were obtained with appropriate consent and institutional review board oversight to allow clinical bridging. Samples must have cfDNA input for LBx testing ≥20 ng DNA for primary analysis, as assessed by the TapeStation assay (RRID: SCR_018435). Exclusion criteria included tissue and other liquid samples, samples that do not meet minimum LBx test operational testing requirements, and samples not obtained with appropriate consent and institutional review board oversight to allow clinical bridging. Samples with <20 ng DNA as assessed by the TapeStation assay were excluded from LBx testing.

**Figure 1:**
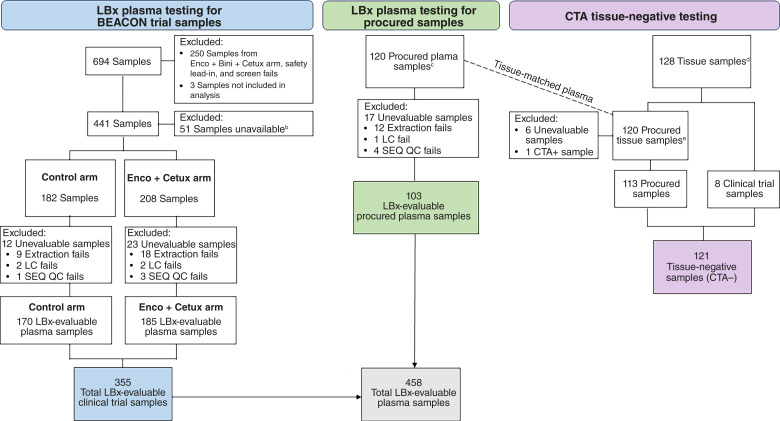
Sample processing flow chart. LC, library construction; QC, quality control; SEQ, sequencing. ^a^Data from these samples are not included in this study. ^b^Plasma sample was not available for testing with LBx. ^c^Matching plasma comes from clinical trial samples. ^d^Tested externally by the Qiagen Therascreen *BRAF* V600E RGQ PCR Kit. ^e^Commercially procured tissue-matched plasma samples that were *BRAF* wild-type by tissue testing and were used for concordance testing.

Specimens included in the clinical bridging study were tested according to the standard testing protocol for the LBx test with recommended cfDNA input of ≥20 ng for the library construction step. Samples for the study were selected from both plasma samples from patients enrolled in the BEACON trial and commercially procured plasma samples from patients with colorectal cancer with matched tissue who were *BRAF*^V600E^ negative. Figure 1 shows the sample disposition for the 694 samples from the 665 patients from the BEACON trial. Samples from the patients randomized to the Enco + Cetux (220 samples) and control (221 samples) arms were selected for analysis; 37 samples from the safety lead-in, 212 samples from the Enco + binimetinib + Cetux arm, three samples from on-treatment blood draws, and one sample from a screen failure were excluded. An additional 51 samples were unavailable for sequencing, and 35 samples with assay failures were excluded, resulting in a total of 355 clinical trial samples (170 control + 185 Enco + Cetux) processed in this bridging study. There were 441 patients with plasma samples from the control and Enco + Cetux arms, consisting of 402 CTA+, 31 CTA unevaluable, and eight CTA− samples. In addition, *BRAF*^V600E^-negative commercial tissue-matched plasma samples from 120 patients not enrolled in the BEACON study were also tested. To mimic the BEACON study enrollment, the 120 commercial tissue samples were tested with the CTA, and the matched plasma samples were subsequently tested with the LBx test. LBx test results for plasma testing were observed for tissue samples that had a negative CTA result.

### Concordance analysis

The concordance between the CTA and the F1LCDx tests was evaluated by the positive percent agreement (PPA) and negative percent agreement (NPA), as well as their two-sided 95% Wilson score CIs. PPA for F1LCDx+/CTA+ was calculated using the formula $PPA=100*\frac{n11}{\left(n11+n10\right)}\%$ and NPA for F1LCDx−/CTA− was calculated using the formula $NPA=100*\frac{n00}{\left(n00+n01\right)}\%$, in which n11 is defined as the number of F1LCDx+/CTA+ samples, n10 is defined as the number of F1LCDx−/CTA+ samples, n00 is defined as the number of F1LCDx−/CTA− samples, and n01 is defined as the number of F1LCDx+/CTA− samples. The prevalence-adjusted positive predictive values (PPV), negative predictive values, and their two-sided 95% bootstrapping CIs were calculated by adjusting for the prevalence of *BRAF*^V600E^ mutations among the intention-to-treat population, with 5% and 10% as the estimated prevalence. The ctDNA TF was quantified using a combination of aneuploidy and variant allele frequencies of genomic alterations, whereas mutations and aneuploidy derived from clonal hematopoiesis were excluded using fragmentomic signal from cfDNA (16, 17).

### Clinical validation

A description of the BEACON study endpoints was previously published (10); details are described in the Supplementary Materials. In the present analysis, the clinical validity of the LBx test was evaluated by assessing clinical efficacy in the F1LCDx *BRAF*^V600E^-positive population (F1LCDx+) based on the ORR difference between the Enco + Cetux and control arms, as well as the log HR between the two arms from the Cox regression model.

### Sensitivity analysis

To assess the robustness of the results subject to missing LBx test results, a sensitivity analysis was performed. Multiple imputation plus bootstrapping were used to impute the F1LCDx *BRAF*^V600E^ status in the LBx-unevaluable population (i.e., no sample was available for testing or the assay failed). The concordance analysis and the clinical efficacy for F1LCDx+ patients were updated by accounting for the imputed data. Multiple imputation was conducted in the original dataset and a total of 50 imputation datasets were generated. The PPA and PPV estimates were computed for each of the 50 imputed complete datasets.

### Statistical analysis

For the concordance analysis results, 95% two-sided CIs were calculated using the Wilson score method for PPA and NPA, whereas they were calculated using the bootstrap method for the adjusted PPV and negative predictive value. For the efficacy results, log HR between the Enco + Cetux and control arms was estimated using the Cox proportional hazards model. For the ORR difference between the Enco + Cetux and control arms, the corresponding 95% two-sided CI was calculated using the Newcombe method.

### Data availability

Upon request, and subject to review, Pfizer will provide the data that support the findings of this study. Subject to certain criteria, conditions, and exceptions, Pfizer may also provide access to the related individual de-identified participant data. See https://www.pfizer.com/science/clinical-trials/trial-data-and-results for more information.

## Results

### Baseline characteristics

Overall, the baseline patient demographics and disease characteristics were similar between patient samples from the LBx-evaluable/CTA+ and LBx-unevaluable/CTA+ subsets (Table 1) and were reflective of the intention-to-treat population in BEACON (10). The representativeness of the analysis population is described in Supplementary Table S1. The median age of patients was 61.0 and 59.5 years in the LBx-evaluable/CTA+ and LBx-unevaluable/CTA+ subsets, respectively. Most patients had Eastern Cooperative Oncology Group performance status of 0 or 1 in both subsets (99.1% in LBx evaluable/CTA+ and 98.7% in LBx unevaluable/CTA+). Most patients had one previous line of therapy (65.9% in LBx evaluable/CTA+ and 64.9% in LBx unevaluable/CTA+).

**Table 1:** Demographics and clinical characteristics in the LBx test-evaluable and -unevaluable subsets

Covariate	CTA+^a^ (*n* = 402)	LBx evaluable/CTA+ (*n* = 328)	LBx unevaluable/CTA+ (*n* = 74)
Age	​	​	​
Median (range), years	61 (27–91)	61 (27–91)	60 (29–80)
Sex, *n* (%)	​	​	​
Female	212 (52.7)	173 (52.7)	39 (52.7)
Male	190 (47.3)	155 (47.3)	35 (47.3)
ECOG PS, *n* (%)	​	​	​
0	197 (49.0)	154 (47.0)	43 (58.1)
1	201 (50.0)	171 (52.1)	30 (40.5)
2	4 (1.0)	3 (0.9)	1 (1.4)
Location of primary tumor, *n* (%)	​	​	​
Both sides	28 (7.0)	24 (7.3)	4 (5.4)
Left colon	137 (34.1)	109 (33.2)	28 (37.8)
Right colon	213 (53.0)	178 (54.3)	35 (47.3)
Unknown colon	24 (6.0)	17 (5.2)	7 (9.5)
Involvement of ≥3 organs, *n* (%)	185 (46.0)	157 (47.9)	28 (37.8)
Presence of liver metastases, *n* (%)	232 (57.7)	194 (59.2)	38 (51.4)
Primary tumor removed, *n* (%)	​	​	​
Completely resected	228 (56.7)	190 (57.9)	38 (51.4)
Partially resected or unresected	174 (43.3)	138 (42.1)	36 (48.7)
Previous lines of therapy, *n* (%)	​	​	​
1	264 (65.7)	216 (65.9)	48 (64.9)
2^b^	137 (34.1)	111 (33.8)	26 (35.1)
High microsatellite instability^c^, *n* (%)	31 (9.5)	25 (8.7)	6 (15.8)
Baseline carcinoembryonic antigen level >5 μg/L, *n* (%)	301 (75.1)	256 (78.1)	45 (61.6)
Baseline C-reactive protein level >10 mg/L, *n* (%)	152 (38.4)	126 (39.0)	26 (35.6)
Irinotecan status, *n* (%)	206 (51.2)	172 (52.4)	34 (46.0)

Abbreviation: ECOG PS, Eastern Cooperative Oncology Group performance status.

a Of the 441 patients in the control and Enco + Cetux arms, 31 were CTA not evaluable and eight were CTA−; therefore, these were excluded, resulting in 402 samples.

b One patient in the control arm received more than two previous lines of therapy.

c High microsatellite instability was determined by PCR.

### Concordance between the CTA and LBx test

To assess the concordance between the CTA and LBx test in detecting *BRAF*^V600E^, a total of 523 CTA-evaluated specimens (402 CTA+ and 121 CTA−) from patients enrolled in the BEACON trial and commercially procured plasma samples were further evaluated. Among the 523 specimens, 481 (360 CTA+ and 121 CTA−) had available plasma samples for LBx testing (Supplementary Table S2). Among the 481 samples analyzed retrospectively by the LBx test, 433 samples (90%) yielded valid F1LCDx testing results. Of the total 402 CTA+ samples, 328 samples were evaluable for the LBx test, with 286 F1LCDx+ and 42 negative for *BRAF*^V600E^ by F1LCDx (F1LCDx−; Table 2). The resulting PPA was 87.2% (95% two-sided Wilson score CI, 83.1%–90.4%; Table 3). Of the 42 cases that were positive for *BRAF*^V600E^ by the tissue-based CTA but not F1LCDx (F1LCDx−/CTA+), 41 (97.6%) had ctDNA TF <1% (Supplementary Table S3). Among samples with ctDNA TF >1%, PPA was 99.4% (95% two-sided Wilson score CI, 96.7%–99.9%) and NPA was 86.7% (95% two-sided Wilson score CI, 62.1%–96.3%; Table 3). Among the patients screened, 105 patients who were negative for *BRAF*^V600E^ by the CTA (CTA−) were included in the concordance analysis (Table 3). Of those, 102 were also found to be *BRAF*^V600E^ negative by the LBx test (F1LCDx−/CTA−) and three were *BRAF*^V600E^ positive by the LBx test (F1LCDx+/CTA−). The resulting NPA was 97.1% (95% two-sided Wilson score CI, 91.9%–99.0%). Among the F1LCDx+/CTA− samples, one (33.3%) had ctDNA TF <1% (Supplementary Table S3).

**Table 2:** Contingency table comparing *BRAF*^V600E^ status between the CTA and F1LCDx

​	CTA+	CTA−	Total
F1LCDx+	286	3	289
F1LCDx−	42	102	144
F1LCDx unevaluable	74	16	90
Total	402	121	523

**Table 3:** Concordance analysis results

​	Prevalence, %	Concordant result with CTA and F1LCDx test	Denominator^a^	Point estimate, (95% two-sided CI^b^), %
PPA	NA	286	328	87.2 (83.1–90.4)
NPA	NA	102	105	97.1 (91.9–99.0)
Adjusted PPV	5	NA	NA	61.6 (40.9–100.0)
Adjusted NPV	5	NA	NA	99.3 (99.1–99.5)
Adjusted PPV	10	NA	NA	77.2 (59.4–100.0)
Adjusted NPV	10	NA	NA	98.6 (98.2–98.9)
ctDNA TF >1%	​	​	​	​
PPA	NA	169	170	99.4 (96.7–99.9)
NPA	NA	13	15	86.7 (62.1–96.3)
Adjusted PPV	5	NA	NA	28.2 (13.6–100.0)
Adjusted NPV	5	NA	NA	100.0 (99.9–100.0)
Adjusted PPV	10	NA	NA	45.3 (24.9–100.0)
Adjusted NPV	10	NA	NA	99.9 (99.7–100.0)

Abbreviations: NA, not available; NPV, negative predictive values.

a The denominator for PPA is the total number of CTA+ samples among the F1LCDx-evaluable samples. The denominator for NPA is the total number of CTA− samples among the F1LCDx-evaluable samples.

b CI was calculated using the Wilson score method for PPA and NPA, whereas for the adjusted PPV and NPV, the bootstrap method was used.

### Clinical validity of the LBx test

We next evaluated the clinical outcomes of patients from the BEACON trial who would have been identified by LBx testing. The median OS of patients who were F1LCDx+/CTA+ (7.6 and 5.4 months in the Enco + Cetux and control arms, respectively; log HR = −0.5) was similar to that of the CTA+ cohort (9.5 and 5.9 months in the Enco + Cetux and control arms, respectively; log HR = −0.5; Table 4). The ORR was similar between patients who were F1LCDx+/CTA+ (18.5% and 1.4% in the Enco + Cetux and control arms, respectively) and in the CTA+ cohort (19.9% and 1.5% in the Enco + Cetux and control arms, respectively; Table 4). The ORR difference (17.1%) between the Enco + Cetux and control arms and the log HR (−0.5) in the F1LCDx+/CTA+ group were similar to those in the CTA+ group (ORR difference, 18.4%; log HR = −0.5). There were 16 responders with complete or partial response who were unevaluable by LBx, resulting in a bigger ORR difference (25.7%) in the F1LCDx-unevaluable/CTA+ population. After imputing the CDx detection status for the LBx-unevaluable samples, the ORR of patients treated with Enco + Cetux improved for the F1LCDx+/CTA+ cohort (mean ORR difference of 18.9% and mean log HR = −0.5) compared with the CTA+ cohort (ORR difference of 18.4%; log HR = −0.5).

**Table 4:** Primary efficacy in the bridging study subpopulations

​	CTA+ (*n* = 402)	F1LCDx+/CTA+ (*n* = 286)	F1LCDx−/CTA+ (*n* = 42)	F1LCDx unevaluable/CTA+ (*n* = 74)
ORR for Enco + Cetux arm, %	19.9	18.5	17.4	28.1
ORR for control arm, %	1.5	1.4	0	2.4
ORR difference (95% two-sided CI),^a^ %	18.4 (12.7–24.6)	17.1 (10.5–24.2)	17.4 (−2.4 to 37.1)	25.7 (9.7–43.1)
Median OS for Enco + Cetux arm, months	9.5	7.6	NA^b^	18.9
Median OS for control arm, months	5.9	5.4	12.2	7.2
Log HR (95% two-sided CI)	−0.5 (−0.8 to −0.3)	−0.5 (−0.8 to −0.2)	−2.7 (−4.7 to −0.7)	−0.4 (−1.2 to 0.3)

Abbreviation: NA, not available.

a CI was calculated using the Newcombe method.

b The estimated median OS is not available because of the small number of events in this group (three events).

Forty-two patients were found to be F1LCDx–/CTA+. The median OS for the F1LCDx–/CTA+ cohort was not available in the Enco + Cetux arm because of the small number of events and 12.2 months in the control arm (log HR = −2.7). The ORR in this cohort was 17.4% and 0.0% in the Enco + Cetux and control arms, respectively. Furthermore, 74 patients were F1LCDx-unevaluable/CTA+. The median OS for this cohort was 18.9 and 7.2 months in the Enco + Cetux and control arms, respectively (log HR = −0.4). The ORR was 28.1% and 2.4% in the Enco + Cetux and control arms, respectively.

### Sensitivity analysis

To assess the robustness of the concordance analysis and efficacy analysis, a sensitivity analysis was performed to account for the missing data for the *BRAF*^V600E^ alteration status in the LBx-unevaluable population. After including imputed data, the estimated median PPA was 85.5%, median PPV (10% prevalence parameter) was 76.9%, and median PPV (15% prevalence parameter) was 84.1% (Supplementary Table S4). For the F1LCDx+/CTA+ population, the median ORR difference after including imputed data was 19.0 (log HR = −0.53; Supplementary Table S5). The estimated efficacy results for the F1LCDx+ population were comparable with those for the CTA+ population (and were better when *c* = 100%; Supplementary Table S6).

## Discussion

Overall, we found a high concordance between the LBx test and the CTA in detecting *BRAF*^V600E^ in samples prospectively collected from patients in the phase III BEACON study, with PPA and NPA values similar to previous concordance analyses between tissue and LBx testing (12, 18–20). The clinical validity of the F1LCDx test in identifying patients with *BRAF*^V600E^ was comparable with that observed in the CTA group, which supports the reasonable assurance of effectiveness of the LBx test. The discordance observed in the F1LCDx−/CTA+ and F1LCDx+/CTA− samples may be attributed to the low ctDNA TF (<1%) among these samples. For samples with low ctDNA TF, concurrent tissue and ctDNA testing should be considered to improve the detection of *BRAF*^V600E^ mutations (18).

The clinical outcomes of patients treated with Enco + Cetux were similar between those in the F1LCDx+/CTA+ cohort and those in the CTA+ cohort. Of note, after imputing the CDx detection status for F1LCDx-unevaluable samples, efficacy for the F1LCDx+/CTA+ population treated with Enco + Cetux improved compared with that of the CTA+ population. These results support the utility of LBx testing for identifying patients with mCRC who have a qualifying *BRAF*^V600E^ mutation and may benefit from treatment with Enco in combination with Cetux.

Patients who are not able to provide evaluable tumor tissue for genomic testing or who face significant health risks from the procedures required to obtain a biopsy may have limited options, and a well-validated and reliable LBx test could provide a pragmatic alternative to tissue biopsy for identifying patients with mCRC who might benefit from Enco in combination with Cetux. The use of multi-gene panels such as F1LCDx enables identification of other alterations within colorectal cancer (e.g., *KRAS*) or across tumor types (e.g., *NTRK1/2/3*) which are relevant to diagnosis and potential treatment options for patients (21, 22). In addition to supporting the identification of patients with *BRAF*^V600E^ mutations who may benefit from treatment with Enco in combination with Cetux, a comprehensive genomic profiling test such as F1LCDx may provide an opportunity for monitoring genomic alterations associated with acquired resistance alterations (23). Furthermore, colorectal cancer is mainly treated in the community, and patients may not be followed systemically in the same center throughout the patient journey. This may require that tissue samples be requested from other institutions, which can be challenging and often lead to delays in testing. LBx offers a more straightforward approach as blood samples can be collected easily and test results can be available to physicians within 2 weeks, leading to appropriate, informed, and personalized treatment strategies for patients. Rapid genomic testing has become increasingly important given the recent approval of Enco, Cetux, and mFOLFOX6, including in the first-line setting, for *BRAF*^V600E^ mCRC.

Although interpretation is limited by the smaller size of the F1LCDx−/CTA+ cohort, a numerically reduced estimated efficacy of Enco + Cetux in the F1LCDx−/CTA+ cohort compared with ORR outcomes in the CTA+ cohort was observed. Additionally, among patients in the F1LCDx−/CTA+ cohort, a numerically improved OS compared with patients in the CTA+ cohort and F1LCDx+/CTA+ cohort was observed. This may be due to a larger tumor bulk needed for detectable ctDNA shedding. Patients with smaller tumor volumes could have better disease prognosis and may thus contribute to the better survival outcomes in the F1LCDx−/CTA+ cohort. This is consistent with the association observed between undetectable or low levels of ctDNA at baseline and/or on treatment and improved PFS and OS across different treatment regimens and tumor types (24–28). To avoid missed detection of *BRAF*^V600E^ mutations, additional tissue testing should be considered if no alterations are detected by the LBx test or if samples have a low ctDNA TF <1% (16, 29, 30). Further investigation is warranted to explore the tumor burden of the disease at baseline in the F1LCDx+/CTA+ and F1LCDx−/CTA+ subsets.

In this retrospective bridging study, the main diagnostic was a tumor tissue–based single gene PCR assay (Qiagen Therascreen), whereas the F1LCDx is an NGS-based comprehensive genomic profiling assay performed on ctDNA extracted from blood. This retrospective bridging was therefore only possible for a subset of participants who had available plasma samples. Additionally, this analysis was not statistically powered.

Overall, this clinical bridging study supports using the LBx test as a clinically valid assay for identifying *BRAF*^V600E^ alterations in patients with mCRC who may be eligible for treatment with Enco in combination with Cetux.

## Supplementary Material

Supplementary Methods Supplementary Methods

Table S1. Representativeness of the patient population analyzed.

Table S2. Plasma sample sizes and sources for LBx testing.

Table S3. Tumor fraction in F1LCDx−/CTA+ and F1LCDx+/CTA− subsets.

Table S4. Summary statistics of PPA and PPV after including imputed data.

Table S5. Summary statistics of estimated log(HR) and ORR difference for the F1LCDx+/CTA+ population (𝜹𝟏) on imputed complete data.

Table S6. Estimated efficacy for the F1LCDx+ population in the sensitivity analysis.

## References

[1] Nikanjam M , Kato S, Kurzrock R. Liquid biopsy: current technology and clinical applications. J Hematol Oncol 2022;15:131. 36096847 10.1186/s13045-022-01351-y PMC9465933

[2] Ranghiero A , Frascarelli C, Cursano G, Pescia C, Ivanova M, Vacirca D, . Circulating tumour DNA testing in metastatic breast cancer: integration with tissue testing. Cytopathology 2023;34:519–29. 37640801 10.1111/cyt.13295

[3] Cheng F , Su L, Qian C. Circulating tumor DNA: a promising biomarker in the liquid biopsy of cancer. Oncotarget 2016;7:48832–41. 27223063 10.18632/oncotarget.9453 PMC5217053

[4] Nakamura Y , Taniguchi H, Ikeda M, Bando H, Kato K, Morizane C, . Clinical utility of circulating tumor DNA sequencing in advanced gastrointestinal cancer: SCRUM-Japan GI-SCREEN and GOZILA studies. Nat Med 2020;26:1859–64. 33020649 10.1038/s41591-020-1063-5

[5] Vellanki PJ , Ghosh S, Pathak A, Fusco MJ, Bloomquist EW, Tang S, . Regulatory implications of ctDNA in immuno-oncology for solid tumors. J Immunother Cancer 2023;11:e005344. 36796877 10.1136/jitc-2022-005344 PMC9936292

[6] Kolenčík D , Shishido SN, Pitule P, Mason J, Hicks J, Kuhn P. Liquid biopsy in colorectal carcinoma: clinical applications and challenges. Cancers (Basel) 2020;12:1376. 32471160 10.3390/cancers12061376 PMC7352156

[7] Bettegowda C , Sausen M, Leary RJ, Kinde I, Wang Y, Agrawal N, . Detection of circulating tumor DNA in early- and late-stage human malignancies. Sci Transl Med 2014;6:224ra24. 10.1126/scitranslmed.3007094 PMC4017867 24553385

[8] Urbini M , Marisi G, Azzali I, Bartolini G, Chiadini E, Capelli L, . Dynamic monitoring of circulating tumor DNA in patients with metastatic colorectal cancer. JCO Precis Oncol 2023;7:e2200694. 37656949 10.1200/PO.22.00694

[9] Chan HT , Chin YM, Low S-K. Circulating tumor DNA-based genomic profiling assays in adult solid tumors for precision oncology: recent advancements and future challenges. Cancers (Basel) 2022;14:3275. 35805046 10.3390/cancers14133275 PMC9265547

[10] Kopetz S , Grothey A, Yaeger R, Van Cutsem E, Desai J, Yoshino T, . Encorafenib, binimetinib, and cetuximab in BRAF V600E-mutated colorectal cancer. N Engl J Med 2019;381:1632–43. 31566309 10.1056/NEJMoa1908075

[11] QIAGEN . QIAGEN launches therascreen BRAF test as companion diagnostic to a BRAFTOVI® (encorafenib) based regimen in metastatic colorectal cancer. [cited [cited 2024 Mar 12]]. Available from: Available from: https://corporate.qiagen.com/newsroom/press-releases/press-release-details/2020/QIAGEN-launches-therascreen-BRAF-test-as-companion-diagnostic-to-a-BRAFTOVI-encorafenib-based-regimen-in-metastatic-colorectal-cancer/default.aspx.

[12] Woodhouse R , Li M, Hughes J, Delfosse D, Skoletsky J, Ma P, . Clinical and analytical validation of FoundationOne Liquid CDx, a novel 324-Gene cfDNA-based comprehensive genomic profiling assay for cancers of solid tumor origin. PLoS One 2020;15:e0237802. 32976510 10.1371/journal.pone.0237802 PMC7518588

[13] NS Medical Devices . FDA approves FoundationOne liquid CDx as CDx for Pfizer’s Braftovi combo in mCRC. [cited 2024 May 23]. Available from: https://www.nsmedicaldevices.com/company-news/fda-approves-foundationone-liquid-cdx-pfizer-braftovi-combo-mcrc/?cf-view.

[14] QIAGEN . therascreen® BRAF V600E RGQ PCR Kit instructions for use (Handbook). Available from: https://www.accessdata.fda.gov/cdrh_docs/pdf19/P190026C.pdf.

[15] Foundation Medicine Inc. FoundationOne®CDx technical information. [cited 2025 May]. Available from: https://www.foundationmedicine.com/media/273/fmi-view.

[16] Rolfo CD , Madison RW, Pasquina LW, Brown DW, Huang Y, Hughes JD, . Measurement of ctDNA tumor fraction identifies informative negative liquid biopsy results and informs value of tissue confirmation. Clin Cancer Res 2024;30:2452–60. 38526394 10.1158/1078-0432.CCR-23-3321 PMC11145175

[17] Chiang AC , Madison RW, Huang Y, Fine A, Jin DX, Oxnard GR, . Abstract 971: validation of a tumor-naïve circulating tumor DNA (ctDNA) response monitoring panel in advanced non-small cell lung cancer (aNSCLC). Cancer Res 2024;84(Suppl 6):971.

[18] Iams WT , Mackay M, Ben-Shachar R, Drews J, Manghnani K, Hockenberry AJ, . Concurrent tissue and circulating tumor DNA molecular profiling to detect guideline-based targeted mutations in a multicancer cohort. JAMA Netw Open 2024;7:e2351700. 38252441 10.1001/jamanetworkopen.2023.51700 PMC10804266

[19] Schulze M , Wang X, Hamad J, Quintanilha JCF, Pasquina LW, Hopkins JF, . Real-world genomic landscape of colon and rectal cancer. FEBS Open Bio 2025;15:674–85. 10.1002/2211-5463.13957 PMC11961397 39865537

[20] Kopetz S , Murphy DA, Pu J, Ciardiello F, Desai J, Van Cutsem E, . Molecular profiling of BRAF-V600E-mutant metastatic colorectal cancer in the phase 3 BEACON CRC trial. Nat Med 2024;30:3261–71. 39313594 10.1038/s41591-024-03235-9 PMC11564101

[21] Mulkidjan RS , Saitova ES, Preobrazhenskaya EV, Asadulaeva KA, Bubnov MG, Otradnova EA, . ALK, ROS1, RET and NTRK1-3 gene fusions in colorectal and non-colorectal microsatellite-unstable cancers. Int J Mol Sci 2023;24:13610. 37686416 10.3390/ijms241713610 PMC10488195

[22] Westphalen CB , Krebs MG, Le Tourneau C, Sokol ES, Maund SL, Wilson TR, . Genomic context of NTRK1/2/3 fusion-positive tumours from a large real-world population. NPJ Precis Oncol 2021;5:69. 34285332 10.1038/s41698-021-00206-y PMC8292342

[23] Woolston A , Barber LJ, Griffiths B, Pich O, Lopez-Bigas N, Matthews N, . Mutational signatures impact the evolution of anti-EGFR antibody resistance in colorectal cancer. Nat Ecol Evol 2021;5:1024–32. 34017094 10.1038/s41559-021-01470-8 PMC7611134

[24] Zhang X , Feng R, Xu Y, Yang L, Xie F, Yang H, . Baseline circulating tumor DNA predicts long-term survival outcomes for patients with early breast cancer. Gland Surg 2024;13:684–96. 38845832 10.21037/gs-24-115 PMC11150192

[25] Pascual J , Gil-Gil M, Proszek P, Zielinski C, Reay A, Ruiz-Borrego M, . Baseline mutations and ctDNA dynamics as prognostic and predictive factors in ER-positive/HER2-negative metastatic breast cancer patients. Clin Cancer Res 2023;29:4166–77. 37490393 10.1158/1078-0432.CCR-23-0956 PMC10570672

[26] Song Y , Hu C, Xie Z, Wu L, Zhu Z, Rao C, . Circulating tumor DNA clearance predicts prognosis across treatment regimen in a large real-world longitudinally monitored advanced non-small cell lung cancer cohort. Transl Lung Cancer Res 2020;9:269–79. 32420066 10.21037/tlcr.2020.03.17 PMC7225135

[27] Zheng Y , Sun H, Cong L, Liu C, Sun Q, Wu N, . Prognostic value of ctDNA mutation in melanoma: a meta-analysis. J Oncol 2021;2021:6660571. 34035810 10.1155/2021/6660571 PMC8116156

[28] Stires H , Zariffa N, Eisele M, Goren E, Espenschied CR, Guha M, . Changes in ctDNA levels as an early indicator of outcomes in advanced NSCLC treated with TKI: initial findings from a retrospective aggregate analysis of 8 clinical trials. J Clin Oncol 2023;41(Suppl 16):3030.

[29] Lin Z , Li Y, Tang S, Deng Q, Jiang J, Zhou C. Comparative analysis of genomic profiles between tissue-based and plasma-based next-generation sequencing in patients with non-small cell lung cancer. Lung Cancer 2023;182:107282. 37392713 10.1016/j.lungcan.2023.107282

[30] Paturu R , Lingaiah R, Kumari N, Singh S, Krishnani N, Srivastava S, . Non-small cell lung cancer: targetable variants in concurrent tissue and liquid biopsy testing in a North Indian cohort. Asian Pac J Cancer Prev 2023;24:3467–75. 37898852 10.31557/APJCP.2023.24.10.3467 PMC10770664

