# Supplementary material for: Clinical Validity of FoundationOne Liquid CDx for Detection of BRAFV600E in Colorectal Cancer
Source: Cancer Res Commun. 2025 Sep 9;5(9):1566–73. doi: 10.1158/2767-9764.CRC-25-0002 (PMC12417970; doi:10.1158/2767-9764.CRC-25-0002)
Supplement: Supplementary Methods [file crc-25-0002_supplementary_methods_suppsm.docx]

# SUPPLEMENTARY METHODS

# *Overview of BEACON study design* *and participants*

The BEACON study design and primary analysis have been published previously(10). Briefly, BEACON (ClinicalTrials.gov: NCT02928224) is a randomized, open-label, phase 3 trial that enrolled patients between May 2017 and January 2019. Patients were randomized 1:1:1 to receive encorafenib (300 mg daily), binimetinib (45 mg twice daily), and cetuximab (400 mg/m^2^ of body-surface area as an initial dose, then 250 mg/m^2^  weekly) (Enco+Bini+Cetux regimen); or encorafenib and cetuximab (Enco+Cetux regimen), administered in the same doses and on the same schedule as for the Enco+Bini+Cetux regimen; or investigators’ choice of either cetuximab (administered in the same doses and on the same schedule as for the other regimens) and irinotecan (180 mg/m^2^ on days 1 and 15) or cetuximab and FOLFIRI (folinic acid [180 mg/m^2^, administered on days 1 and 15], fluorouracil [400 mg/m^2^ as an initial dose, then 1200 mg/m^2^/day for 2 days, initiated on days 1 and 15], and irinotecan [at the same dose and on the same schedule as the other regimens]) (control group)(10). Randomization was stratified according to Eastern Cooperative Oncology Group performance status (0 vs 1), previous use of irinotecan (yes vs no), and cetuximab formulation (US-licensed vs European-approved formulation).

# *Clinical validation*

The primary endpoints were overall survival (OS) and objective response rate (ORR) in the Enco+Bini+Cetux group compared with the control group, as assessed by independent central review(10). ORR is defined as the proportion of patients with objective response of either confirmed complete response or partial response based on Response Evaluation Criteria in Solid Tumors 1.1. Secondary endpoints included OS in the Enco+Cetux arm compared with the control arm, as well as progression-free survival, duration of response, and safety in all arms (10).
