# Supplementary material for: Clinical Validity of FoundationOne Liquid CDx for Detection of BRAFV600E in Colorectal Cancer
Source: Cancer Res Commun. 2025 Sep 9;5(9):1566–73. doi: 10.1158/2767-9764.CRC-25-0002 (PMC12417970; doi:10.1158/2767-9764.CRC-25-0002)
Supplement: Table S1. — Representativeness of the patient population analyzed. [file crc-25-0002_table_s1.suppst1.docx]

**Table S1.** Representativeness of the patient population analyzed.

| Cancer type | BRAF V600E-mutant metastatic colorectal cancer |
| --- | --- |
| Considerations related to: |  |
| Sex and gender | Despite colorectal cancer being more common in males overall, *BRAF* V600E mutations are more frequent among females. |
| Age | Most patients with colorectal cancer are diagnosed aged 50 years or older; *BRAF* V600 mutations are more prevalent in elderly patients. |
| Geography | Incidence rates are higher in transitioned countries compared with transitioning countries. The world regions with the highest incidence rates are Europe, Australia/New Zealand, and North America. |
| Other considerations | *BRAF* V600 mutations are more common in right-sided colon cancer. |
| Overall representativeness  of the analysis population in this study | In our analysis group of patients with metastatic colorectal cancer positive for the *BRAF* V600E mutation (n=402), a subset of patients from the global phase 3 BEACON trial, there was a slight majority of females (52.7%). Median age was 61 years (range 27–91). The majority of primary tumors were located in the right colon (53.0%). |
