# Supplementary material for: Clinical Validity of FoundationOne Liquid CDx for Detection of BRAFV600E in Colorectal Cancer
Source: Cancer Res Commun. 2025 Sep 9;5(9):1566–73. doi: 10.1158/2767-9764.CRC-25-0002 (PMC12417970; doi:10.1158/2767-9764.CRC-25-0002)
Supplement: Table S2. — Plasma sample sizes and sources for LBx testing. [file crc-25-0002_table_s2.suppst2.docx]

**Table S2.** Plasma sample sizes and sources for LBx testing.

| Plasma sample sources | CTA+  (n=360) | CTA−  (n=121) |
| --- | --- | --- |
| BEACON trial | 360^a^ | 8 |
| Enco+Cetux arm | 190 | 3 |
| Control arm | 170 | 5 |
| Commercially procured tissue-matched | NA | 113^b^ |

CTA, clinical trial assay; Enco+Cetux, encorafenib + cetuximab; F1LCDx, FoundationOne^®^Liquid CDx; LBx, liquid biopsy; NA, not available.

The totals reflect all available plasma samples for F1LCDx assessment, including both evaluable (i.e., successful sequencing) and unevaluable (i.e., assay failure). ^a^Three hundred and sixty plasma samples were from 402 CTA+ samples in the BEACON trial (subtracting 31 CTA-unevaluable samples and 8 CTA− samples from 441 clinical trial samples in total). ^b^Seven commercially procured samples (six CTA-unevaluable samples and one CTA+ sample) were excluded from this study.
