# Supplementary material for: Clinical Validity of FoundationOne Liquid CDx for Detection of BRAFV600E in Colorectal Cancer
Source: Cancer Res Commun. 2025 Sep 9;5(9):1566–73. doi: 10.1158/2767-9764.CRC-25-0002 (PMC12417970; doi:10.1158/2767-9764.CRC-25-0002)
Supplement: Table S3. — Tumor fraction in F1LCDx−/CTA+ and F1LCDx+/CTA− subsets. [file crc-25-0002_table_s3.suppst3.docx]

**Table S3.** Tumor fraction in F1LCDx−/CTA+ and F1LCDx+/CTA− subsets.

| Tumor fraction | F1LCDx−/CTA+  (n=42) | F1LCDx+/CTA−  (n=3) |
| --- | --- | --- |
| <1% | 41 | 1 |
| >1% | 1 | 2 |

CTA, clinical trial assay; F1LCDx, FoundationOne^®^Liquid CDx.
