# Supplementary material for: Clinical Validity of FoundationOne Liquid CDx for Detection of BRAFV600E in Colorectal Cancer
Source: Cancer Res Commun. 2025 Sep 9;5(9):1566–73. doi: 10.1158/2767-9764.CRC-25-0002 (PMC12417970; doi:10.1158/2767-9764.CRC-25-0002)
Supplement: Table S4. — Summary statistics of PPA and PPV after including imputed data. [file crc-25-0002_table_s4.suppst4.docx]

**Table S4.** Summary statistics of PPA and PPV after including imputed data.

|  | PPA, % | PPV, % | PPV, % |
| --- | --- | --- | --- |
| Prevalence parameter | NA | 10% | 15% |
| Mean (min, max) | 85.5  (84.3, 86.8) | 76.9  (76.6, 77.1) | 84.1  (83.9, 84.3) |
| 2.5% | 84.5 | 76.7 | 83.9 |
| Q1 | 85.0 | 76.8 | 84.0 |
| Median | 85.5 | 76.9 | 84.1 |
| Q3 | 85.8 | 76.9 | 84.1 |
| 97.5% | 86.5 | 77.1 | 84.2 |

max, maximum; min, minimum; NA, not available; PPA, positive percent agreement; PPV, positive predictive values;
Q, quartile.
