# Supplementary material for: Clinical Validity of FoundationOne Liquid CDx for Detection of BRAFV600E in Colorectal Cancer
Source: Cancer Res Commun. 2025 Sep 9;5(9):1566–73. doi: 10.1158/2767-9764.CRC-25-0002 (PMC12417970; doi:10.1158/2767-9764.CRC-25-0002)
Supplement: Table S5. — Summary statistics of estimated log(HR) and ORR difference for the F1LCDx+/CTA+ population (𝜹𝟏) on imputed complete data. [file crc-25-0002_table_s5.suppst5.docx]

**Table S5.** Summary statistics of estimated log(HR) and ORR difference for the

F1LCDx+/CTA+ population (𝜹𝟏) on imputed complete data.

|  | ORR difference, % | Log (HR) |
| --- | --- | --- |
| Mean (min, max) | 18.9 (17.9, 19.8) | −0.5 (−0.6, −0.5) |
| 2.5% | 17.9 | −0.6 |
| Q1 | 18.6 | −0.6 |
| Median | 19.0 | −0.5 |
| Q3 | 19.3 | −0.5 |
| 97.5% | 19.7 | −0.5 |

CTA, clinical trial assay; F1LCDx, FoundationOne^®^Liquid CDx; HR, hazard ratio; max, maximum; min, minimum;
ORR, objective response rate; Q, quartile.
