# Supplementary material for: Clinical Validity of FoundationOne Liquid CDx for Detection of BRAFV600E in Colorectal Cancer
Source: Cancer Res Commun. 2025 Sep 9;5(9):1566–73. doi: 10.1158/2767-9764.CRC-25-0002 (PMC12417970; doi:10.1158/2767-9764.CRC-25-0002)
Supplement: Table S6. — Estimated efficacy for the F1LCDx+ population in the sensitivity analysis. [file crc-25-0002_table_s6.suppst6.docx]

**Table S6.** Estimated efficacy for the F1LCDx+ population in the sensitivity analysis.

| c^a^ | 𝜹_𝑪𝑫𝒙+_ with 95% CI [log (HR)] | 𝜹_𝑪𝑫𝒙+_ with 95% CI  (ORR difference) |
| --- | --- | --- |
| Prevalence parameter = 10% | | |
| 30% | −0.4 [−0.7, −0.2] | 15.9 [9.8, 21.9] |
| 50% | −0.5 [−0.7, −0.2] | 16.7 [10.7, 22.7] |
| 70% | −0.5 [−0.8, −0.2] | 17.6 [11.5, 23.7] |
| 100% | −0.5 [−0.8, −0.2] | 18.9 [12.5, 25.3] |
| Prevalence parameter = 15% | | |
| 30% | −0.5 [−0.7, −0.2] | 16.8 [10.8, 22.9] |
| 50% | −0.5 [−0.8, −0.2] | 17.4 [11.3, 23.5] |
| 70% | −0.5 [−0.8, −0.2] | 18.0 [11.8, 24.2] |
| 100% | −0.5 [−0.8, −0.2] | 18.9 [12.5, 25.3] |

CI, confidence interval; HR, hazard ratio; ORR, objective response rate.

^a^c is the ratio of efficacy between F1LCDx+/CTA− and F1LCDx+/CTA+ populations.
